# SLC39A14 Is a Potential Therapy Target and Prognostic Biomarker for Acute Myeloid Leukemia

**DOI:** 10.3390/genes16080887

**Published:** 2025-07-27

**Authors:** Yun Li, Liming Shan

**Affiliations:** 1 Department of Hematology, Affiliated Hangzhou First People’s Hospital, School of Medicine, Westlake University, Hangzhou 310006, China; 2 Team of Neonatal & Infant Development, Health and Nutrition, NDHN, School of Biology and Pharmaceutical Engineering, Wuhan Polytechnic University, Wuhan 430024, China; liyunez@163.com

**Keywords:** acute myeloid leukemia, programmed cell death, SLC39A14, prognosis, immune

## Abstract

**Background**: Programmed cell death-related genes (PCDRGs) have been reported to play an important role in diagnosis, treatment and immunity regarding cancer, but their prognostic value and therapeutic potential in acute myeloid leukemia (AML) patients still need to be fully explored. **Methods**: Cox regression analysis and Least Absolute Shrinkage and Selection Operator (LASSO) analysis were used to identify PCDRGs significantly associated with the prognosis of AML patients. Furthermore, a prognostic risk model for AML patients was constructed based on the selected PCDRGs, and their immune microenvironment and biological pathways were analyzed. Cell experiments ultimately confirmed the potential role of PCDRGs in AML. **Results**: The results yielded four PCDRGs that were used to develop a prognostic risk model, and the prognostic significance of this model was confirmed using an independent external AML patient cohort. This prognostic risk model provides an independent prognostic risk factor for AML patients. This prognostic feature is related to immune cell infiltration in AML patients. The inhibition of solute carrier family 39 member 14 (SLC39A14) expression enhanced apoptosis and inhibited cell cycle progression in AML cells. **Conclusions**: This study integrates bioinformatics analysis and cellular experiments to reveal potential gene therapy targets and prognostic gene markers in AML.

## 1. Introduction

Acute myeloid leukemia (AML) is a hematological cancer characterized by the abnormal development and proliferation of myeloid precursor cells. This condition leads to the buildup of these cells in the bone marrow and bloodstream [1]. Currently, the most common subtype of leukemia worldwide is AML, which exhibits high heterogeneity in clinical practice [2]. Approximately 487,000 new leukemia cases were diagnosed worldwide in 2022, ranking it as the second most common blood cancer, with 305,000 deaths, the highest mortality rate among hematological cancers [3]. Advances in comprehending the molecular diversity and disease mechanisms of AML have led to notable enhancements in treatment approaches. These include refined cytotoxic chemotherapy, the development of targeted therapies, optimized hematopoietic stem cell transplantation protocols, and the integration of immunotherapeutic strategies [4]. With a deeper understanding of the molecular heterogeneity and pathogenesis of acute myeloid leukemia, significant progress has been made in its treatment, mainly including improvements in traditional cytotoxic chemotherapy and targeted drugs, the standardization of hematopoietic stem cell transplantation, and the application of immunotherapy. However, the 5-year overall survival rate for patients under 60 years old is only 40%, while, for patients over 60 years old, it is 5–15%. In addition, patients who relapse after complete remission rarely survive for more than 5 years [5]. Consequently, it is crucial to investigate the pathogenesis and therapeutic targets of AML to enhance patient prognosis.

Programmed cell death (PCD) is reported to be significant in cancer development [6,7]. PCD can occur through multiple mechanisms, such as anoikis, apoptosis, alkaliptosis, autophagy, lysosome-dependent cell death, cuproptosis, NETosis, entosis, immunogenic cell death, entotic cell death, ferroptosis, pyroptosis, necroptosis, methuosis, oxeiptosis, parthanatos, and paraptosis [8]. Apoptosis is characterized by a series of biochemical and structural changes in cells. Pyroptosis, another form of PCD, is initiated by inflammasome activation and involves caspase-1, leading to cell swelling, membrane disruption, and the release of inflammatory cytokines. Autophagy, essential for cellular homeostasis, degrades damaged components and can promote either cell survival or death, depending on the context. Necroptosis, resembling necrosis, is mediated by RIPK1 and RIPK3 activation. Cuproptosis, induced by excessive copper, results in lipid peroxidation and mitochondrial impairment. Entotic cell death, unique to living cells and their vicinities, bypasses traditional apoptotic pathways. Parthanatos is a regulated cell death form driven by the overactivation of PARP-1 nuclease. Lysosome-mediated cell death results from the leakage of hydrolases into the cytosol, caused by membrane damage. Alkaliptosis, a novel PCD form, is regulated by intracellular alkalinization. Oxeiptosis, a newly discovered pathway associated with KEAP1’s oxidative stress response, is likely interconnected with other cell death processes [9,10].

In AML, tumor cells employ various strategies to evade the activation of programmed cell death mechanisms. They can alter signaling pathways or change their morphologies to evade host clearance [11]. These findings highlight the importance of PCD research in advancing our understanding of AML and in the development of novel anti-AML therapies. Previous studies have shown that PCD-related genes (PCDRGs) hold therapeutic potential for various cancers [12,13]. The function of these PCDRGs in AML remains unclear. In this work, we systematically analyzed the prognostic value of PCDRGs in AML patients. We conducted consensus clustering, signature construction, and functional enrichment analysis of PCDRGs; investigated their association with the AML immune microenvironment; and validated the potential significance of PCDRGs in AML through cellular experiments. These analyses enhance the prognostic evaluation system and offer a new therapeutic approach for AML.

## 2. Materials and Methods

### 2.1. AML Dataset Preprocessing

This study utilized two independent datasets, TARGET-AML and GSE37642, comprising a total of 323 AML samples. Gene expression levels were processed and evaluated using R version 4.4.1 [14]. Our analysis utilized 1547 key regulatory genes (PCDRGs) [10].

### 2.2. Identification of PCDRGs with Prognostic Relevance in AML Patients

The survvcutpoint function of the survminer package (based on the R version 4.4.1) was used to determine the optimal expression threshold for each PCDRG in the TARGET-AML dataset and GSE37642 dataset. Based on the optimal expression threshold of each PCDRG, AML patients were stratified into a prognostic high-expression group and a prognostic low-expression group. Meanwhile, univariate Cox regression analysis was used to evaluate the correlation between each PCDRG and the overall survival rate of AML patients. The Kaplan–Meier curve was used to calculate the prognostic differences for each PCDRG. The statistical significance was analyzed using the log-rank test (*p* < 0.05). The Least Absolute Shrinkage and Selection Operator (LASSO) method, implemented via the glmnet package, was used to identify the most predictive genes for survival outcomes in AML patients. The optimal penalty parameter minimizing the prediction error was identified using ten-fold cross-validation [15].

### 2.3. Consensus Clustering Analysis of Survival-Related PCDRGs

Based on the TARGET-AML dataset, the ConsensusClusterPlus package in R was utilized to perform consensus clustering, aiming to investigate the functional and prognostic significance of the selected PCDRGs. The analysis involved 1000 iterations and evaluated a cluster range with k = 10 to identify the optimal clustering solution. Principal component analysis was used to confirm the number of clusters obtained based on the consensus clustering analysis of four PCDRGs. Finally, the Kaplan–Meier curve of the optimal number of clusters was used to evaluate the prognostic significance [16].

### 2.4. Construction and Validation of AML Prognostic Risk Score Model

Based on the TARGET-AML dataset, a prognostic scoring formula was developed using multivariable Cox regression coefficients, expressed as risk score = β1 × Exp1 + β2 × Exp2 + βi × Exp, where β denotes the coefficients and Exp signifies the expression levels of the chosen genes. AML patients were divided into high-risk and low-risk prognostic groups using the median risk scoring formula. Meanwhile, Kaplan–Meier analysis and log-rank tests were used to analyze the prognostic survival differences between different risk groups, and the prognostic significance of this risk model was validated in the GSE37642 dataset [17].

### 2.5. Biological Pathway Analysis

Based on the TARGET-AML dataset, biological pathway analysis was conducted on genes significantly associated with the prognostic risk scores (| R | > 0.3) of AML patients. The R package was employed for Gene Ontology (GO), Kyoto Encyclopedia of Genes and Genomes (KEGG) pathway, and gene set enrichment analysis (GSEA) assessments. FDR < 0.2 and *p*-value < 0.05 were considered as significance thresholds [18].

### 2.6. Analysis of Variations in Tumor Immune Cell Infiltration Across Distinct Risk Groups

Based on the TARGET-AML dataset, the CIBERSORT package (R software package) was used to analyze the immune microenvironment between AML patients in the high-risk and low-risk prognostic groups. The difference in immune cell infiltration between the low-risk prognosis group and high-risk prognosis group was calculated using the Wilcoxon rank sum test. The correlation among immune cell infiltration and PCDRGs was calculated using the Spearman method [19].

### 2.7. Estimation of Therapeutic Drugs

Based on the TARGET-AML dataset, the oncoPredict R package was utilized to evaluate the sensitivity of 198 different drugs in AML. The study aimed to identify drugs exhibiting notable IC50 value differences between the low-risk and high-risk groups [20].

### 2.8. SLC39A14 Interference in Constructed AML Cell Model

HL-60 cells were sourced from the cell bank at the First People’s Hospital of Hangzhou, Zhejiang Province. Cells were transfected with three designed siRNAs targeting SLC39A14. HL-60 cells were seeded on a 24-well plate (2 × 10^5^ cells per well) and then cultured for 24 h. Subsequently, they were transfected with either one of three types of siRNA (HL-60+SLC39A14-siRNA1 (59), HL-60+SLC39A14-siRNA2 (515), HL-60+SLC39A14-siRNA3 (1052)) or a negative control (HL-60+NC-siRNA) containing a random sequence. Interference with a highly efficient silencing target gene (GAPDH) was used as a positive control (positive controls are used to confirm the reliability of the transfection process and detection methods). Reverse-transcription quantitative PCR (RT-qPCR) was used to evaluate the interference efficiency, leading to the selection of the most effective siRNA for further experiments [21]. Each group had three replicates, and an ordinary one-way ANOVA was performed for multi-group comparative analysis. *p* < 0.05 was considered significant.

### 2.9. Apoptosis Experiment

Transfected HL-60 cells, blank control group cells (blank control refers to HL-60+NC-siRNA cell samples without any fluorescent markers added—this control was used to set the background signal of the flow cytometer and adjust the instrument settings to eliminate spontaneous fluorescence and other non-specific signals), HL-60+NC-siRNA group cells, and HL-60+SLC39A14-siRNA2 (515) group cells (1 × 10^6^ cells/time) were harvested and washed twice with pre-cooled PBS and then centrifuged at 300× *g* at 4 °C for 5 min each time. Then, 1 × Binding Buffer was added and the cells were adjusted to the same concentration (1 × 10^6^/mL). Then, 1–2 × 10^5^ cell suspensions were placed in a flow cytometer, an appropriate amount of Annexin V-FITC labeled with fluorescent dye and an appropriate amount of propidium iodide (PI) were added, and they were gently mixed. This was incubated at room temperature in the dark for 15–20 min. Cell viability and apoptosis were evaluated by flow-cytometric analysis within one hour post-staining. Student’s *t*-test (unpaired) was used to check the statistical significance while comparing the means of two groups; each group had three replicates. *p* < 0.05 was considered significant [22]. The specifications of the flow cytometer used in the apoptosis experiment and the relevant names and sources of the reagents are listed in Figure S1.

### 2.10. AML Cell Cycle Experiment

Transfected HL-60 cells (HL-60+NC-siRNA group cells and HL-60+SLC39A14-siRNA2 (515) group cells) were collected and centrifuged at 200× *g* for 2 min to remove the supernatant, and they were washed with 1 mL of 1 × PBS. The HL-60 cells were then resuspended and fixed in 0.5 mL of pre-cooled 70% ethanol at 4 °C for more than 30 min. After centrifugation at 200× *g* for 2 min, the ethanol was removed and cells were washed three times with 1 mL of 1 × PBS. Cells were then resuspended in a 50 μg/mL PI staining solution and incubated at 37 °C for 30 min before flow-cytometric analysis. Student’s *t*-test (unpaired) was used to check the statistical significance while comparing the means of two groups; each group had three replicates. *p* < 0.05 was considered significant [23]. The specifications of the flow cytometer used in the cell cycle experiment and the relevant names and sources of the reagents are listed in Figure S1.

### 2.11. Statistical Analysis

To compare the two variables, we used *t*-tests or Wilcoxon tests. To evaluate survival differences, Kaplan–Meier curves and log-rank tests were employed. Costimulatory molecular genes were evaluated for their prognostic value using univariate and multivariate Cox regression models. To assess variations in the distribution of clinical variables in DLBC patients, Pearson’s chi-squared test was utilized. The R program was used for all of the methods involved in this investigation. The Spearman analysis method was used for correlation analysis. For cell experiments, Student’s *t*-test was used for comparisons between the two groups. Ordinary one-way ANOVA was used for multi-group comparative analysis. The threshold for statistical significance was *p* < 0.05 [22].

## 3. Results

### 3.1. Identification of PCDRGs with Prognostic Relevance in AML Patients

**Figure 1:**
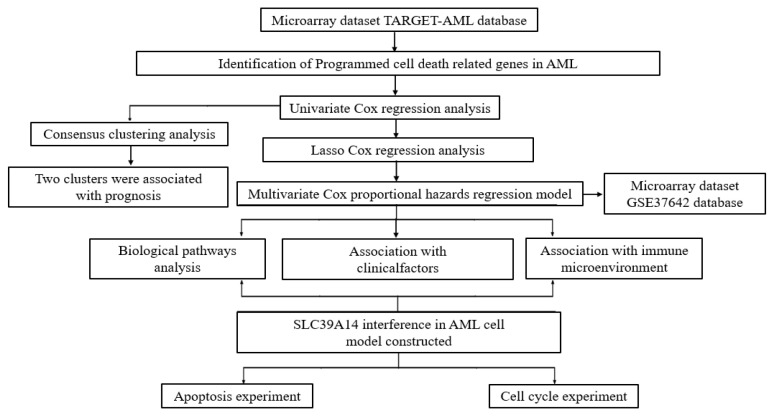
The flowchart of the present study design.

This study’s workflow is shown in Figure 1. There were 76 PCDRGs with significant differences (*p* < 0.05) in the prognostic stratification of AML patients. The LASSO analysis method was used to further screen for PCDRGs with significant differences in prognostic stratification, and the prognostic differences among the screened PCDRGs were validated in the GSE37642 dataset.

**Figure 2:**
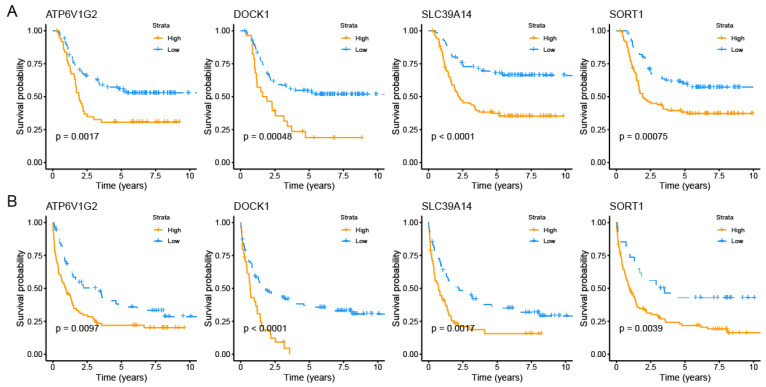
Survival analysis for four PCDRGs. The Kaplan–Meier curves confirmed each gene’s prognostic value in the TARGET-AML dataset (**A**). The Kaplan–Meier curves confirmed each gene’s prognostic value in the GSE37642 dataset (**B**).

Finally, we obtained four PCDRGs with prognostic significance (Figures S2 and S3). The Kaplan–Meier curves confirmed the significant prognostic differences among four PCDRGs in AML patients (Figure 2A,B). In AML, elevated levels of SLC39A14, ATP6V1G2, DOCK1, and SORT1 correlate with unfavorable outcomes.

### 3.2. Consensus Clustering Analysis Based on Prognostic Significance of PCDRGs

**Figure 3:**
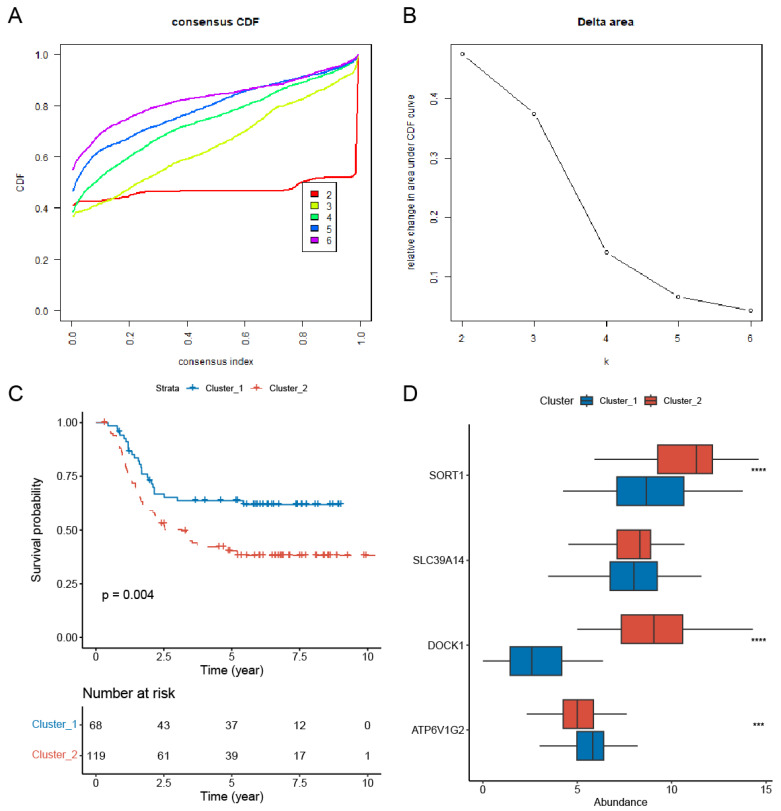
Consensus clustering analysis of prognostic survival-related PCDRGs. (**A**) The cumulative distribution function of consensus clustering from k = 2–6. (**B**) The relative change in the area under the CDF curve from k = 2–6. (**C**) AML patients in cluster 1 had a better prognosis than those in cluster 2. (**D**) Compared to cluster 1, DOCK1 and SORT1 were significantly upregulated in cluster 2. *** *p* < 0.001, **** *p* < 0.0001.

We performed consistent clustering analysis. Our results indicated that classifying patients into two clusters (k = 2) yielded more stable groupings compared to higher numbers of clusters (k = 3–6) (Figure 3A,B). This finding was further corroborated by PCA graphs (Figure S4). The prognostic significance of the two-cluster classification was assessed using Kaplan–Meier survival analysis. The findings indicated a marked difference in outcomes, with patients in cluster 2 showing a significantly poorer prognosis than those in cluster 1 (Figure 3C). DOCK1 and SORT1 were significantly upregulated in cluster 2, as shown in Figure 3D.

### 3.3. Construction and Validation of AML Prognostic Risk Score Model

**Figure 4:**
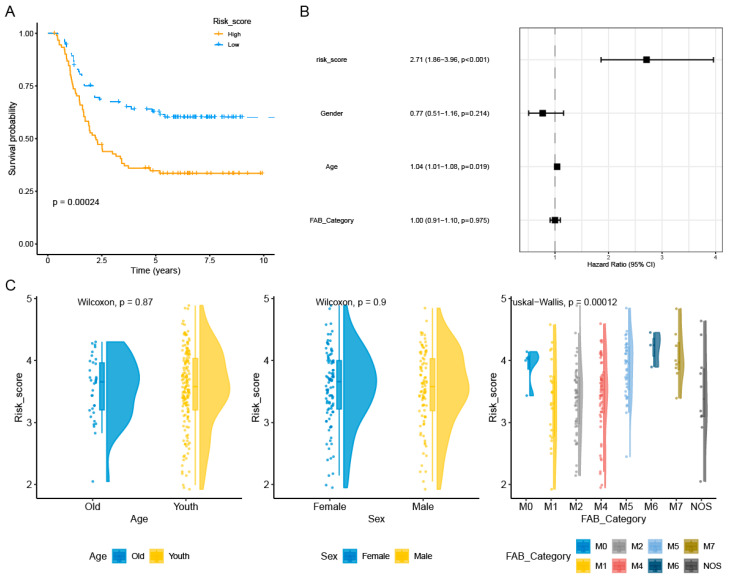
Relationship between the prognostic model and clinical pathological factors of AML patients. Kaplan–Meier curves showed that AML patients with a high risk had a worse prognosis compared with those with a low risk (**A**). The risk score was identified as an independent risk factor for the prognosis of AML patients by multivariate Cox regression analysis (*p* < 0.01 represents significance) (**B**). We also found that AML patients with FAB_Category had different risk scores (*p* < 0.01 represents significance) (**C**).

The expression and coefficients of the four selected PCDRGs (SLC39A14, ATP6V1G2, DOCK1, and SORT1) and their transcription levels were used to construct a prognostic risk score for AML patients. The construction formula for the risk score is as follows: risk score = 0.2338 × ATP6V1G2 + 0.0861 × DOCK1 + 0.0840 × SLC39A14 + 0.1081 × SORT1. AML patients were stratified into a prognostic high-risk group and a prognostic low-risk group based on this median risk scoring formula. Meanwhile, our Kaplan–Meier survival analysis showed that high-risk AML patients have poorer prognostic outcomes (Figure 4A). This result was further validated using the independent GSE37642 dataset (Figure S5). Multivariate Cox regression analysis revealed that this prognostic risk score is an independent prognostic factor for AML patients (Figure 4B). We also found that there were differences in risk scores among AML patients with FAB_Category (Figure 4C). We evaluated the AML patients’ risk score predictions for the 1-year, 5-year, and 8-year mortality rates, finding AUC values of 0.69, 0.74, and 0.68 in the TARGET-AML dataset and 0.68, 0.75, and 0.76 in the GSE37642 dataset (Figure S6). Therefore, our risk scoring model can effectively support clinical practice by enhancing diagnosis and treatment.

### 3.4. Biological Pathways

**Figure 5:**
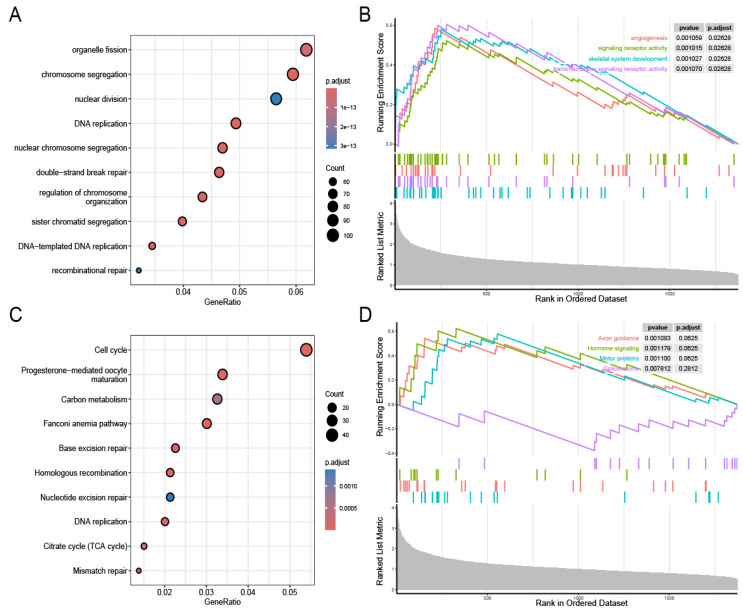
Identification of prognostic model-related biological pathways. GO functional analysis (**A**). GSEA-GO functional analysis (**B**). KEGG functional analysis (**C**). GSEA-KEGG functional analysis (**D**).

In total, 2169 positively correlated genes were screened for biological pathway analysis. Figure 5A,B present the biological pathway analysis results for the GO and KEGG assessments. The GO analysis results mainly indicated enrichment in chromosome segregation and DNA replication, while the KEGG analysis results mainly indicated enrichment in the cell cycle. The GSEA indicated that high-risk patients were predominantly characterized by angiogenesis and signaling receptor activity (Figure 5C,D).

### 3.5. Variations in Tumor Immune Cell Infiltration Across Distinct Risk Groups and Their Association with Drug Efficacy

**Figure 6:**
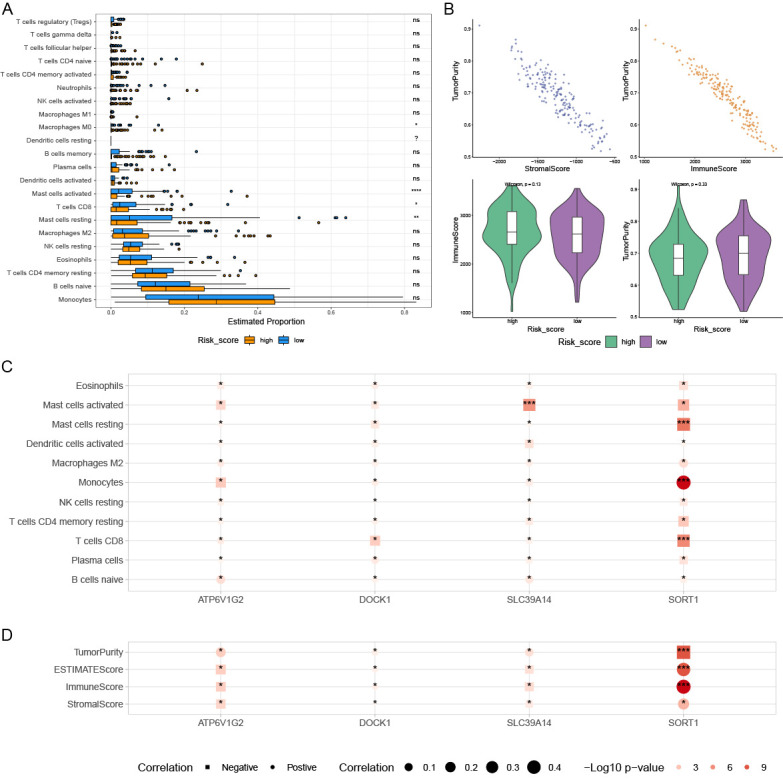
The relationships within the tumor immune microenvironment. The box plots show a significant difference in immune cell infiltration and immune score between low-risk patients and high-risk patients, and the differences in various immune cells’ proportions are complex (**A**,**B**). High-risk patients had higher tumor purity (**B**). Four prognostic PCDRGs are also significantly associated with immune cells in AML (**C**,**D**). * *p* < 0.05, ** *p* < 0.01, *** *p* < 0.001, **** *p* < 0.0001, ns *p* ≥ 0.05.

**Figure 7:**
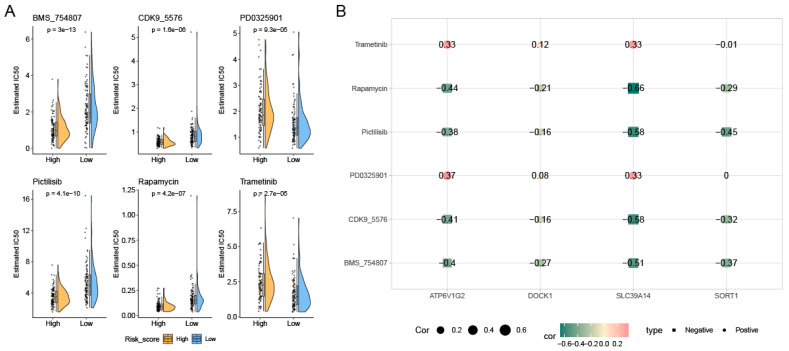
Drug sensitivity analysis. We identified 6 drugs with significant differences in IC50 between the low-risk and high-risk groups (**A**). The expression of SLC39A14 was negatively correlated with the drug sensitivity of Rapamycin, Pictilisib, CDK9_5576, and BMS_754807 (**B**).

Figure 6A,B illustrate the significant differences in immune cell infiltration and immune scores between the prognostic high-risk group and prognostic low-risk group, highlighting the complexity in the proportions of various immune cells. Four PCDRGs show a significant association with immune cells in AML (Figure 6C,D). We assessed the drug sensitivity of 198 drugs in AML using the oncoPredict R software package. Six drugs exhibited significant IC50 differences between the low-risk and high-risk groups: BMS_754807, CDK9_5576, PD0325901, Pictilisib, Rapamycin, and Trametinib (Figure 7A). The expression of SLC39A14 was negatively correlated with the drug sensitivity of Rapamycin, Pictilisib, CDK9_5576, and BMS_754807 (Figure 7B).

### 3.6. SLC39A14 Interference in Constructed AML Cell Model

qPCR analysis indicated that SLC39A14 interference was most pronounced in the HL-60+SLC39A14-siRNA2 (515) group compared to the control group (HL-60+NC-siRNA) cells (Figure S7). Therefore, the AML cells in the HL-60+SLC39A14-siRNA2 (515) group were used as an AML cell model.

### 3.7. Apoptosis Experiment

**Figure 8:**
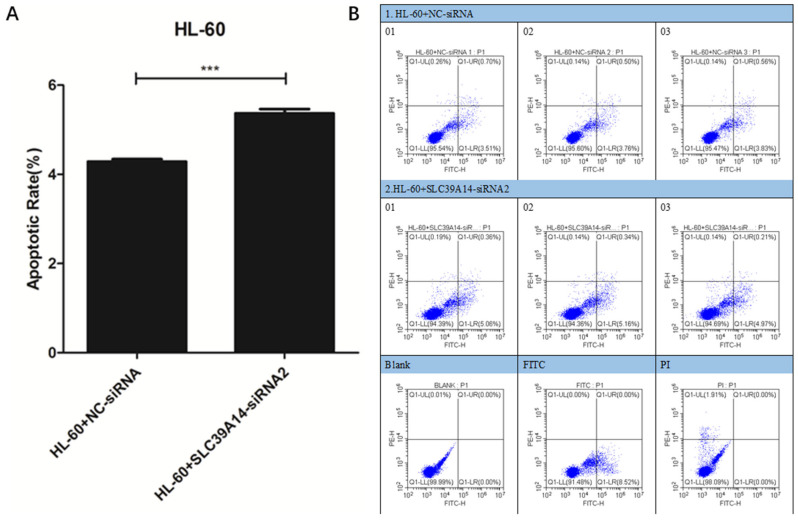
Apoptosis experiment. Cell apoptosis results showed that, compared with the control group (HL-60+NC-siRNA), the AML apoptosis rate of the HL-60+SLC39A14-siRNA2 (515) group cells was increased (**A**,**B**). *p* < 0.05 was considered significant. *** *p* < 0.001.

The results of cell apoptosis indicated an increase in the apoptosis rate of AML cells in the HL-60+SLC39A14-siRNA2 (515) group compared to the control group (HL-60+NC-siRNA) (Figure 8A,B).

### 3.8. Cell Cycle Experiment

**Figure 9:**
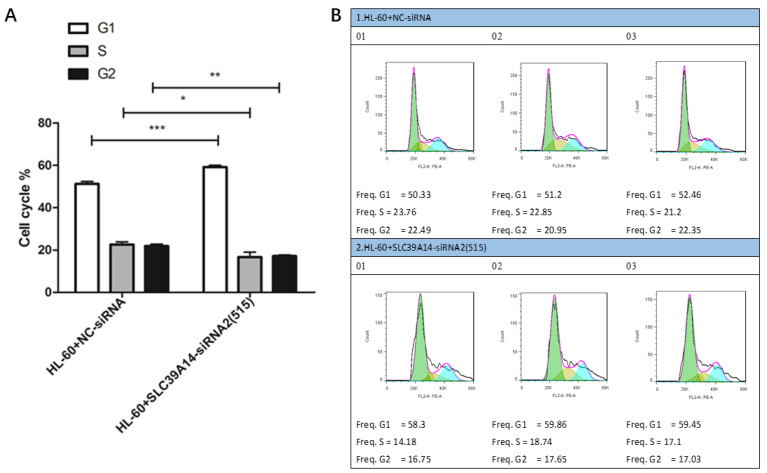
Cell cycle experiment. Cell cycle results showed that, compared with the control group (HL-60+NC-siRNA), the HL-60+SLC39A14-siRNA2 (515) group showed the significant inhibition of AML cells in the G1 phase (**A**,**B**). *p* < 0.05 was considered significant. * *p* < 0.05, ** *p* < 0.01, *** *p* < 0.001.

Cell cycle analysis indicated that the HL-60+SLC39A14-siRNA2 (515) group exhibited inhibitory effects on AML cells in the G1 phase compared to the control group (HL-60+NC-siRNA) (Figure 9A,B).

## 4. Discussion

At present, research on prognostic markers for AML patients is being deepened. Advances in various fields, such as cytogenetics, molecular genetics, epigenetics, the immune microenvironment, and metabolic markers, provide an important basis for the precise treatment and prognostic evaluation of AML patients. An increasing number of prognostic markers for AML patients are being discovered, which are of great significance in the diagnosis, treatment selection, drug monitoring, and prognostic evaluation of AML patients [24]. Cytogenetic abnormalities are an important basis for the prognostic evaluation of AML patients. According to the chromosomal karyotype, AML patients can be divided into low-risk, moderate-risk, and high-risk groups [25,26]. Molecular genetic markers are becoming increasingly important in the prognostic stratification of AML patients, especially for acute myeloid leukemia with normal cytogenetics. FLT3 internal tandem repeat mutations are associated with high white blood cell counts, high recurrence rates, and poor overall survival in AML patients. NPM1 mutations are usually associated with a better prognosis in AML patients, especially in the absence of FLT3-ITD mutations. The double-allele mutation of CEBPA is associated with a better prognosis in AML patients, especially in young AML patients. TP53 mutations are associated with complex karyotypes and a poor prognosis in AML patients. ASXL1 mutations are associated with a poor prognosis in AML patients, especially in elderly AML patients. The DNMT3A mutation is common in AML patients, especially in elderly patients with a poor prognosis. Minimal residual disease refers to a small amount of leukemia cells remaining after treatment, which is an important predictor of recurrence in AML patients [27,28]. Epigenetic changes play an important role in the occurrence and progression of acute myeloid leukemia, with DNA methylation and histone modification being current research hotspots. TET2 mutations are associated with abnormal DNA methylation, often leading to a poor prognosis in AML patients. EZH2 mutations are associated with abnormal histone modifications, leading to a poor prognosis in AML patients [29,30]. The immune microenvironment in acute leukemia patients plays an important role in disease progression and treatment response. The infiltration of immune checkpoint molecules and immune cells (such as T cells, NK cells) may affect the prognosis of AML patients [31]. The metabolic reprogramming of acute myeloid leukemia cells, such as glycolysis and oxidative phosphorylation, is associated with disease progression and drug resistance. Elevated levels of metabolic markers such as LDH are associated with a poorer prognosis in AML patients [32]. Although these prognostic markers have played a positive role in the treatment of AML in clinical practice, the prognosis is still poor. Therefore, the discovery of new prognostic markers for AML has significant therapeutic implications for AML patients [33]. The biological pathways associated with programmed cell death play an important role in the development of cancer, and it has been reported that PCDRGs are associated with the progression of various cancers [34]. In this study, the value of PCDRGs in AML patients was evaluated, and a prognostic risk model for AML patients was ultimately established based on four PCDRGs. This model does not only provide an independent prognostic factor for AML patients but is also related to the immune microenvironment and clinical characteristics of AML patients. Therefore, this predictive model provides new insights for the clinical diagnosis and treatment of AML patients.

The primary objective of various tumor immunotherapies has consistently been to induce apoptosis in tumor cells [35]. Immune cells can induce cancer cell apoptosis by delivering granzymes or activating death ligands. Research indicates that apoptotic lymphoma cells may enhance tumor growth and inhibit immune responses against tumors [36,37]. In AML cells, it has been confirmed that the expression of SLC39A14 can promote apoptosis. Our study identified PCDRGs as crucial in regulating the immune response in AML, offering a theoretical foundation for AML immunotherapy.

In AML patients, the high expression of SLC39A14 leads to a poor prognosis. Currently, no research has documented the significance of SLC39A14 in AML. SLC39A14 significantly influences cancer prognosis, cellular function, and pathological mechanisms [38]. SLC39A14 could play a crucial role in AML progression by influencing intracellular regulation. Cell experiments have confirmed that SLC39A14 regulates the apoptosis and cycle progression of AML cells. SLC39A14 represents a promising therapeutic target for AML.

This research inevitably has several limitations. This retrospective study, primarily based on GEO databases, may have been affected by confounding factors, necessitating further investigation through prospective research. The study demonstrates that SLC39A14 expression modulates AML cell function. In vitro cell experiments lack the interactions present in vivo and do not account for neuroendocrine system regulation. In vitro experiments also do not completely capture the properties of the tissue of origin. Consequently, additional animal experiments are necessary in future research.

## 5. Conclusions

This study identified molecular subtypes based on PCDRGs in AML patients and constructed a prognostic signature. In addition, the immune infiltration, biological pathways, and drug prediction of different risk groups were considered. This signature may contribute to the clinical evaluation of AML patients’ prognosis. Cell experiments have confirmed that SLC39A14 regulates the cycle progression and apoptosis of AML cells. SLC39A14 may be a potential target for AML treatment.

## Data Availability

The microarray dataset (GSE37642) was downloaded from the Gene Expression Integrated Database (GEO) (available online: https://www.ncbi.nlm.nih.gov/geo/, accessed on 6 January 2025). The TARGET-AML dataset can be downloaded from the Therapeutically Applicable Research to Generate Effective Treatments (TARGET) database (https://www.cancer.gov/ccg/research/genome-sequencing/target, accessed on 6 January 2025).

## Supplementary Materials

The following supporting information can be downloaded at: https://www.mdpi.com/article/10.3390/genes16080887/s1, Figure S1. The model of the flow cytometer used in the cell experiment and the relevant names and sources of purchase of the reagents were listed. Figure S2. The least absolute shrinkage and selection operator (LASSO) Cox regression analysis. Figure S3. The multivariate Cox regression analyses of four PCD-related genes. Figure S4. Identification of cluster numbers using consensus clustering. Figure S5. Kaplan-Meier curves for risk scores from the GSE37642 dataset. Figure S6. The risk score predictions for 1-year, 5-year and 8-year and 10-year mortality rates. Figure S7. SLC39A14 interference in AML cell model constructed.
