# Supplementary material for: SLC39A14 Is a Potential Therapy Target and Prognostic Biomarker for Acute Myeloid Leukemia
Source: Genes (Basel). 2025 Jul 27;16(8):887. doi: 10.3390/genes16080887 (PMC12385646; doi:10.3390/genes16080887)
Supplement: Supplementary file 1 [file genes-16-00887-s001.zip › genes-3733229-supplementary.pdf]

Experimental instruments for apoptosis experiments

Name	Purchase source	Model
flow cytometer	Beckman	Beckman Cytoflex

The main experimental reagents for apoptosis experiments

Name	Purchase source
RPMI-1640	Hyclone
FBS	LONSERA
Apoptosis assay kit	Solarbio

Experimental instruments for cell cycle experiments

Name	Purchase source	Model
flow cytometer	Beckman	Beckman Cytoflex

The main experimental reagents for cell cycle experiments

Name	Purchase source
RPMI-1640	HyClone
FBS	Gibco
Propidine iodide	Solarbio

Figure S1 The model of the flow cytometer used in the cell experiment and the relevant names and sources of purchase of the reagents were listed

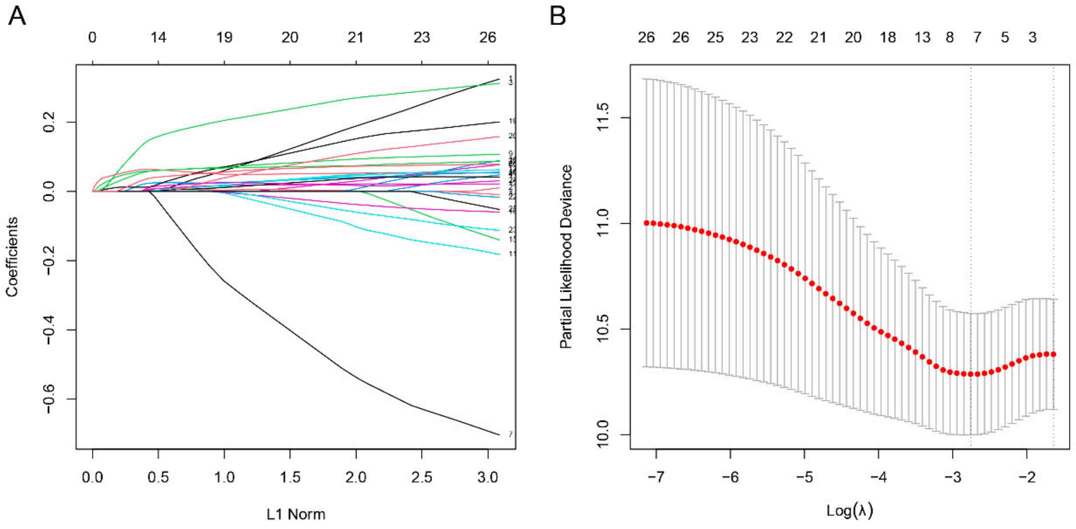

Figure S2 The least absolute shrinkage and selection operator (LASSO) Cox regression analysis

Plot of the coefficients of LASSO Cox regression analysis (A). The plot of partial likelihood deviance in the TARGET_AML dataset (B).

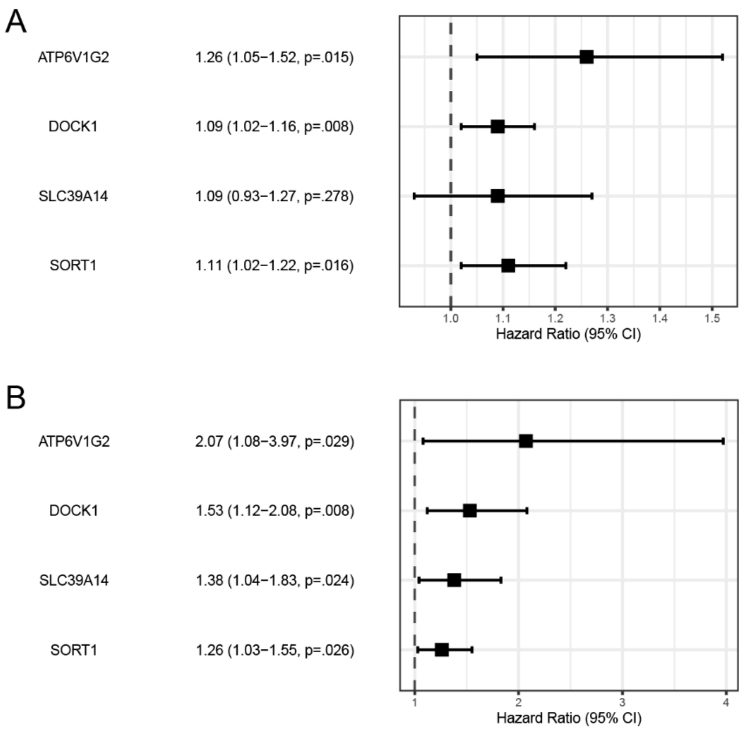

Figure S3 The multivariate Cox regression analyses of four PCD-related genes

The multivariate Cox regression analyses of four PCD-related genes in the TARGET_AML dataset (A). The multivariate Cox regression analyses of four PCD-related genes in the GSE37642 dataset (B).

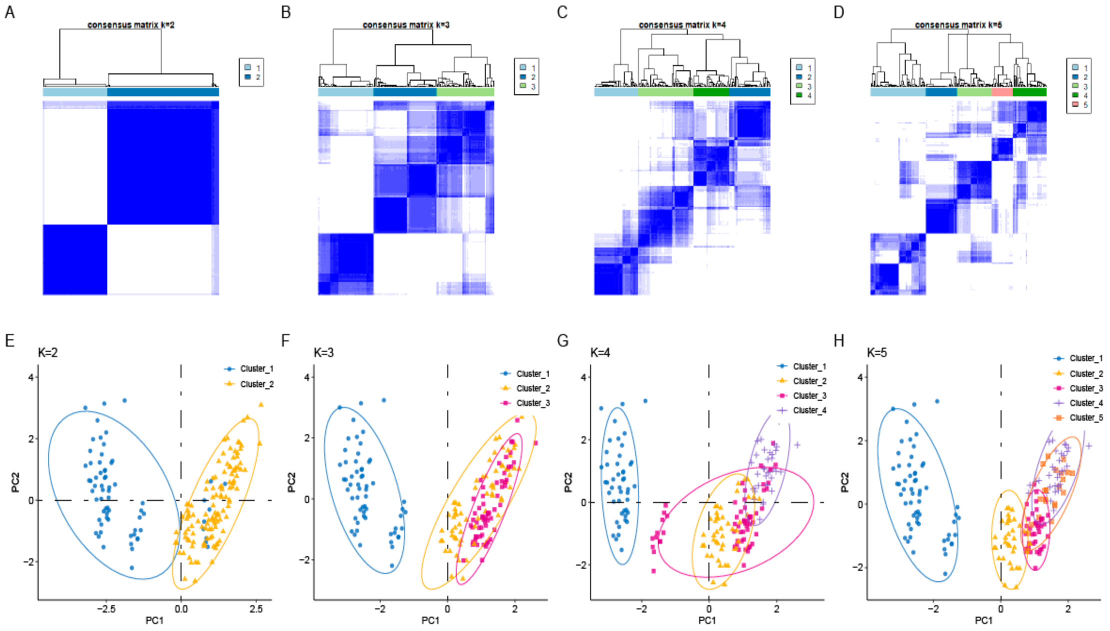

Figure S4 Identification of cluster numbers using consensus clustering

Consensus clustering matrix for k = 2 (A), k = 3 (B), k = 4 (C), and k = 5 (D). Principal component analysis for evaluating the distributions of different cluster numbers (E-H). The different coloured points show the different clusters.

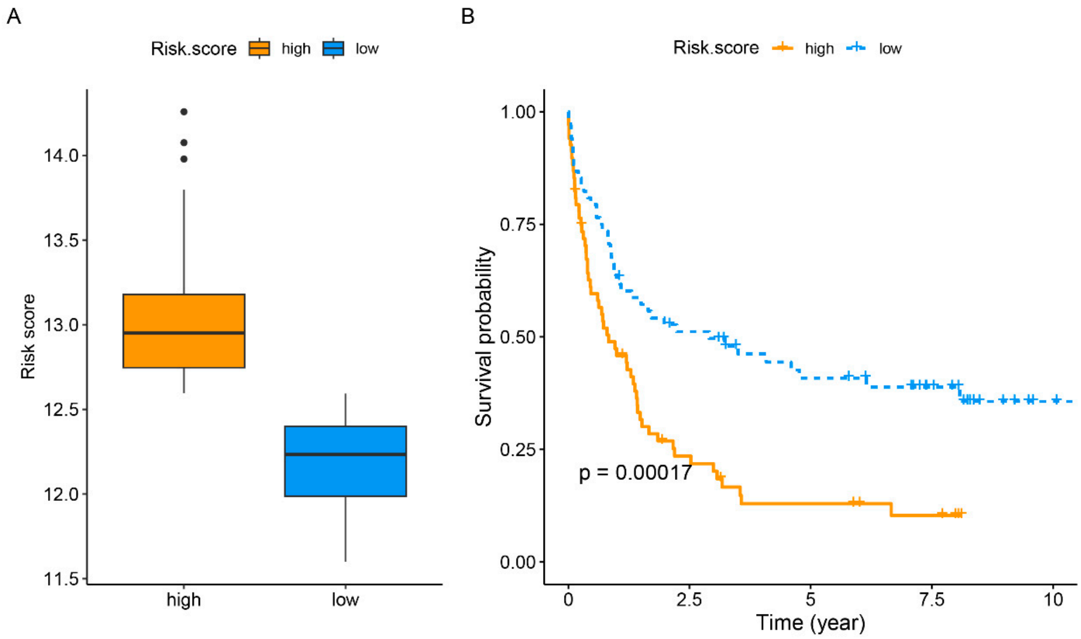

Figure S5 Kaplan-Meier curves for risk scores from the GSE37642 dataset

Orange, representing the high-risk scores; blue, representing the low-risk scores (A).

Orange, representing the high-risk scores; blue, representing the low-risk scores (B).

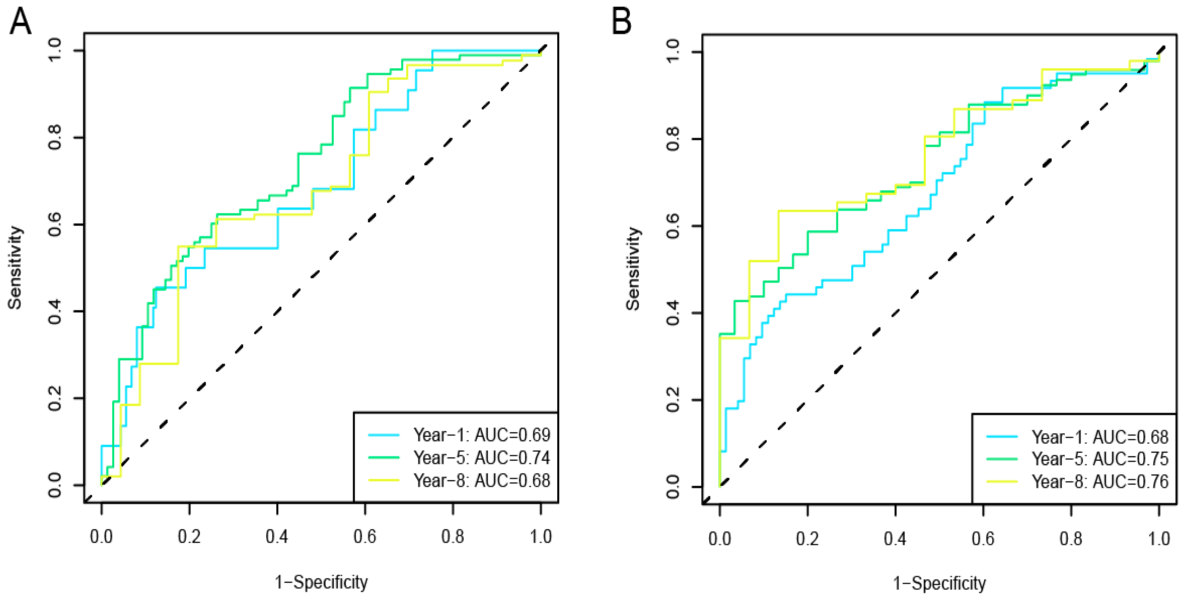

Figure S6 The risk score predictions for 1-year, 5-year and 8-year and 10-year mortality rates
We also examined the risk score predictions for 1-year 5-year and 8-year mortality rates and found that the area under the receiving work characteristic curve (AUC) values in TARGET_AML dataset were 0.69, 0.74 and 0.68 (A) and in the GSE37642 dataset were 0.68, 0.75 and 0.76, respectively (B).

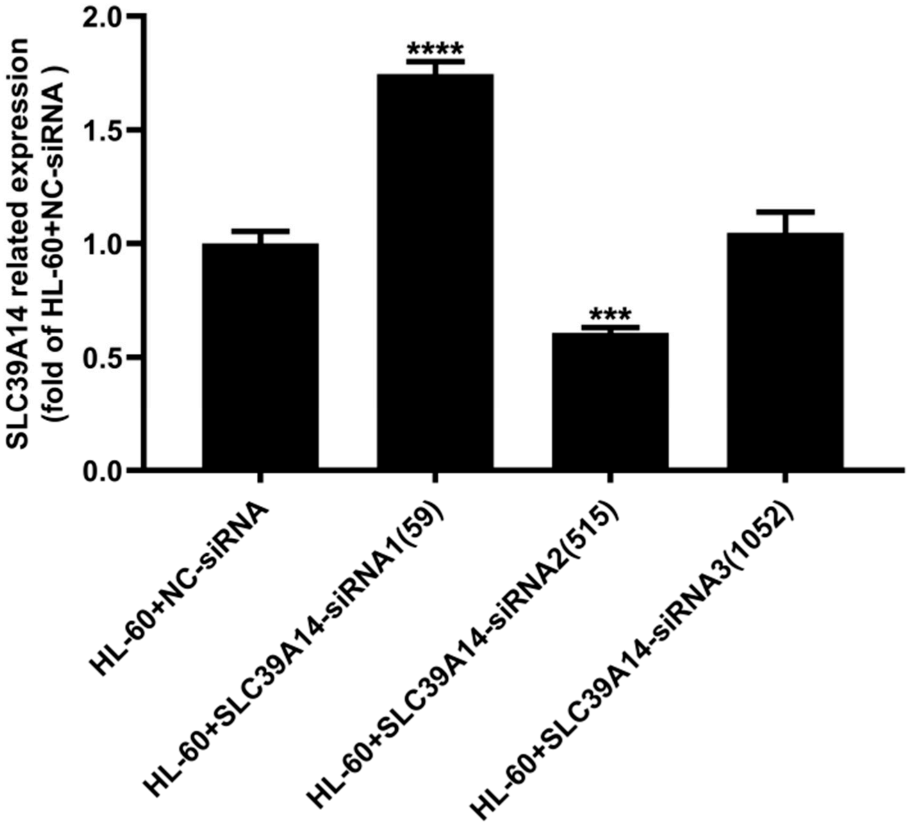

Figure S7 SLC39A14 interference in AML cell model constructed

The qPCR results showed that compared with the control group (HL-60+NC-siRNA) cells, the interference effect of SLC39A14 was most significant in the HL-60+SLC39A14-siRNA2(515) group. Therefore, the AML cells in the HL-60+SLC39A14-siRNA2(515) group were used as a AML cell model. *** $p < 0.001$, **** $p < 0.0001$
